# Machine learning chained neural network analysis of oxygen transport amplifies the physiological relevance of vascularized microphysiological systems

**DOI:** 10.1002/btm2.10582

**Published:** 2023-08-01

**Authors:** James J. Tronolone, Tanmay Mathur, Christopher P. Chaftari, Yuxiang Sun, Abhishek Jain

**Affiliations:** ^1^ Department of Biomedical Engineering, College of Engineering Texas A&M University College Station Texas USA; ^2^ Department of Nutrition, College of Agriculture and Life Sciences Texas A&M University College Station Texas USA; ^3^ Department of Medical Physiology, School of Medicine Texas A&M Health Science Center Bryan Texas USA; ^4^ Department of Cardiovascular Science Houston Methodist Academic Institute Houston Texas USA

**Keywords:** artificial intelligence, cell transplant, islet, machine learning, microphysiological system, vascularization

## Abstract

Since every biological system requires capillaries to support its oxygenation, design of engineered preclinical models of such systems, for example, vascularized microphysiological systems (vMPS) have gained attention enhancing the physiological relevance of human biology and therapies. But the physiology and function of formed vessels in the vMPS is currently assessed by non‐standardized, user‐dependent, and simple morphological metrics that poorly relate to the fundamental function of oxygenation of organs. Here, a chained neural network is engineered and trained using morphological metrics derived from a diverse set of vMPS representing random combinations of factors that influence the vascular network architecture of a tissue. This machine‐learned algorithm outputs a singular measure, termed as vascular network quality index (VNQI). Cross‐correlation of morphological metrics and VNQI against measured oxygen levels within vMPS revealed that VNQI correlated the most with oxygen measurements. VNQI is sensitive to the determinants of vascular networks and it consistently correlates better to the measured oxygen than morphological metrics alone. Finally, the VNQI is positively associated with the functional outcomes of cell transplantation therapies, shown in the vascularized islet‐chip challenged with hypoxia. Therefore, adoption of this tool will amplify the predictions and enable standardization of organ‐chips, transplant models, and other cell biosystems.

**Translational Impact Statement**

There is an unmet need to robustly design and standardize the vascularization of microphysiological systems. The purpose of this work was to derive a quantitative evaluation metric that describes the biological function of vascular network, that is, transporting oxygen to its tissue. Deep learning chained neural networks were made to generate a vascular network quality index (VNQI), that were trained with oxygen distribution heatmaps calculated from images. VNQI is a physiologically relevant, automated, and standardized determinant than conventional measures, and it can optimize preclinical translational biosystems.

## 1 INTRODUCTION

Blood capillaries are central to the survival and function of almost every living cell, tissue, or organ. Vascular networks made of capillaries are crucial because they transport the oxygen and nutrition needed for sustaining life. Therefore, to better understand human biology, it may be desirable to design, engineer, and evaluate preclinical models of such, cell‐, tissue‐, and organ‐level tissue systems with vascularized networks. In the practice of regenerative medicine, also the success of almost every cell transplant depends on the speed at with which vascularization may occur in the tissue. For example, pancreatic islet transplantation therapy is a growing treatment strategy for type I diabetes (T1D), but in many cases, it suffers from difficulties initiating the growth and fenestration of oxygenating capillary beds, the absence of which would eventually result in islet death via hypoxia.^1^ Therefore, objective and quantitative assessment of vascularization potential and its long‐term benefits in preclinical models of human physiology, pathophysiology, and even cell transplantation therapies are needed.

Microphysiological systems (MPS), also known as organs‐on‐chip, are one of the most contemporary in vitro platform technologies used to model preclinical science, drugs and chemical interventions, and medicinal products.^2^ While most of the models that were developed in the previous decade or so lacked perfused and functional vascular networks,^3^, ^4^ more recent advances have begun to include their development and function.^3^, ^4^, ^5^, ^6^, ^7^ The vascularized MPS (vMPS) and cell biosystems will continue to grow and become a mainstream technology, but there is still an unmet need to derive and apply an evaluative and standardized metric of measurement for the vascularized networks in these systems. Through a recent review of vascularized biological model systems, we discovered the primary determinants of vascularization (cell source and density, biophysical elements, biochemical supplements), where prior studies of angiogenesis or vasculogenesis on‐chip focused on a better understanding of how these determining factors regulate network formation and function.^8^ However, we also found that the measurement of angiogenesis and vasculogenesis in these systems was made via a widely varied selection of one of several vascular morphological metrics. Recently, we applied several incrementally complex supervised statistical and machine learning algorithms and showed that many of these metrics are inconsistent with each other, user‐dependent, and poorly correlate to the oxygen transport phenomena of vascular networks when applied independently or in combination.^9^ While some groups have also directly measured perfusion capacity of networks with fluorescent beads,^10^, ^11^ this experimental strategy can be extremely difficult in practice as some capillaries might be smaller than the beads (tracer particles are typically orders of magnitude larger than oxygen molecules). Further, these particles do not diffuse into extravascular regions like oxygen, thus limiting the reliability of their measure and physiological‐relevance to mass transport.

Here, we employ deep learning to analyze a highly diverse set of vMPS that were engineered through various combinations of the mechanical and chemical factors influencing the vascular network architecture of any tissue. We designed a chained neural network that is trained using images of vMPS and a variety of commonly used and easily measured morphological metrics. Our algorithm outputs a singular measure, termed as vascular network quality index (VNQI), that can be applied to a broad range of vMPS. We compared VNQI to independently used metrics relative to measured oxygen within the residing tissue, repeatedly finding that VNQI is a physiologically relevant metric that can be used in a variety of preclinical investigations where vascular function is included. Finally, to validate the algorithm in a proof‐of‐concept islet transplant model that could treat T1D, we fabricated vascularized islet‐chips and demonstrated that VNQI may be applied as a scoring criterion to design cell transplant models and predict their performance.

## 2 METHODS

### 2.1 Dataset generation

All 500 images used in this study were obtained from vMPS systems whose detailed fabrication, cell culture, immunostaining, and image processing are reported earlier. Briefly, vMPS were fabricated using traditional photo‐ and soft‐lithography practices, where polydimethylsiloxane (PDMS) pre‐polymer was cast on molds containing the chip design, baked, processed, and bonded to glass slides. Chips were left in an oven overnight to render them hydrophobic, and subsequently UV treated to sterilize them before cell culture. Various ingredients (hydrogels, cells, growth factors, etc.) were injected into their respective compartments and chips were cultured for 96 h with daily media changes. The rationale for choosing a 96 h timepoint in the context of this work is that at the 96‐h mark, we were able to obtain a highly diverse set of 500 images of the vascular networks that could be used to derive the VNQI. Chips were then fixed and imaged, resulting in tiled and z‐stacked image files that were then orthogonally projected and cropped for image processing. The vessel morphological metrics were calculated based on published literature.^12^ We used our own AngioMT software to measure oxygen transport phenomena in the vMPS systems, quantified as a normalized area average oxygen concentration within the intravascular region, termed OXY_*V*_, and the extravascular region, termed OXY_*T*._ ^13^ OXY_V_ and OXY_*T*_ values for this study were calculated with AngioMT using two workstations: (1) Mac Mini (Apple) having a 3.0 GHz, 6‐core Intel Core i5 processor, 32 GB RAM, a 256 GB solid state hard drive, and an Intel UHD Graphics 6301536 MB graphics card; (2) OptiPlex 780 (Dell) having a 3.16 GHz, 2‐core Intel Core 2 processor, 16 GB RAM, a 500 GB solid state hard drive, and an ATI Radeon HD 3450 graphics card.

### 2.2 Machine learning and VNQI calculation

Machine learning experiments were carried out using Python 3.8.8 via Anaconda3. All code was written in JupyterLab 3.0.14 web browser. Morphological datasets from the previously published REAVER^12^ tool were modified with additional columns for the OXY_*V*_ and OXY_*T*_ measurements corresponding to each sample and used as input for all analyses. First, exploratory data analyses were carried out to determine the distribution of all variables. Here, we determined that based on their distributions as well as their dependencies on other morphological metrics, the following REAVER metrics were excluded from further analyses: vessel length density, mean tortuosity, mean valency, and maximum diffusion distance.

Data for machine learning were split using scikit‐learn^14^ train_test_split (), which separated the *x* and *y* data into a training set containing 80% of the data and a testing set containing the other 20%. Neural network regression was accomplished utilizing AutoKeras,^15^ an open‐source, peer reviewed auto machine learning tool that optimizes neural network hyperparameters such as the number of hidden layers and nodes. StructuredDataRegressor from autokeras was used, which outputted an optimal neural network regression model depending on the specified network input architectures. Accuracy metrics were determined using scikit‐learn metrics (r2_score, mean_absolute_error, and mean_squared_error). Following the determination of an optimal neural network architecture, all samples from the database were sent through the framework, generating VNQIs for all samples which were used for downstream analyses.

All plots generated from the Python scripts were exported as SVG files using pyplot.savefig from matplotlib^16^ and developed into publication‐ready figures using Adobe Illustrator.

### 2.3 Vascularized islet‐chip design

100X islet media supplements were prepared by dissolving HEPES, l‐glutamine, and Na‐pyruvate in 400 mL basal RPMI‐1640 media (without l‐glutamine; 45000‐404, VWR) to final concentrations of 10, 2, and 1 mM, respectively. 2‐mercaptoethanol (M3148‐25ML, Sigma Aldrich) was added to the supplement mix at a final concentration of 0.05 mM and the entire mixture was adjusted to pH 7.2 before aliquoting and storing at −20°C. 5 mL of this supplement was added to 500 mL RPMI‐1640 (without l‐glutamine) with 10% fetal bovine serum (FBS) and 1% 100X antibiotic–antimycotic. INS‐1E insulinoma cells as reported previously were expanded in T75 flasks with 25 mL media refreshed every 48 h.^17^, ^18^ Islets were lifted at confluency following the same lifting procedure as described earlier and adjusted to a concentration of 20 million islets/mL. Islets were stained using PKH26 live cell tracking kit (MINI26‐1KT, Sigma Aldrich) according to the manufacturer's protocol. Following staining, the cells were adjusted to a concentration of 0.3 million islets/mL. Cells were then seeded into an AggreWell 400 6‐well spheroid formation plate (34,450, StemCell Technologies; Vancouver, BC, V6A 1B6, Canada) that had been prepared according to the manufacturer's protocol and incubated for 48 h. After spheroid formation, islets were harvested according to the manufacturer's protocol and resuspended in a 2X fibrinogen solution before mixing with thrombin‐containing EC solutions. Upon mixing 1:1, EC + Islet solutions were injected into the central channel and solidified before following the same vasculogenesis‐chip culturing protocol. For EC‐Islet coculture experiments, 1:1 INS‐media:EGM‐2 was used. A schematic detailing of the cell pre‐culturing, gel encapsulation, and device compartmentalization is shown in Figure S1.

### 2.4 Insulin secretion

After 96 h, vascularized islet‐chips were imaged to determine the number of islets in each chip. Islets were later counted using ImageJ's Analyze Particles macro. Immediately following imaging, vascularized islet‐chips received fresh media and were placed in a hypoxia incubator where the O_2_ concentration was set to 5% for 4 h. Next, each chip received new media containing 10 mM glucose before returning to the hypoxia chamber for 1 h. Effluent media was collected from the fluid channel outlets and replaced with a fibrin‐digestion solution containing 50 fibrinolytic units nattokinase (NATT10, BulkSupplements; Henderson, NV 89011, USA) and 1 mM EDTA (BP2482100, Fisher Scientific) in 1X PBS.^19^ Devices were left on a shaker set to 100 rpm for 30 min, and the digested solution containing the remaining insulin as well as cells were centrifuged for 5 min at 500 rcf. The resulting supernatant along with a cell pellet containing endothelial cells and islets were collected, as well as LFs in some groups. The supernatants were analyzed for insulin content using a pre‐coated insulin ELISA kit (ELR‐Insulin‐1, RayBioTech; Corners, GA 30092, USA). Absorbance measurements were carried out using a BioTek Cytation 5 plate reader (Agilent, Santa Clara, CA 95051, USA). To normalize the insulin measurements, each measurement was divided by the total number of spheroids in each sample.

### 2.5 Statistics

All statistical analysis was done in GraphPad Prism 9. Student's *t*‐tests were used with two‐group experiments and ordinary one‐way ANOVA with Tukey post hoc tests were used for experiments with three groups or more. All data are represented as mean ± standard error of the mean. Significance is represented as *p < 0.05; **p < 0.01; ***p < 0.001; ****p < 0.0001. Insignificant comparisons are left unannotated.

## 3 RESULTS

### 3.1 Design and evaluation strategy of vascularized networks

**FIGURE 1:**
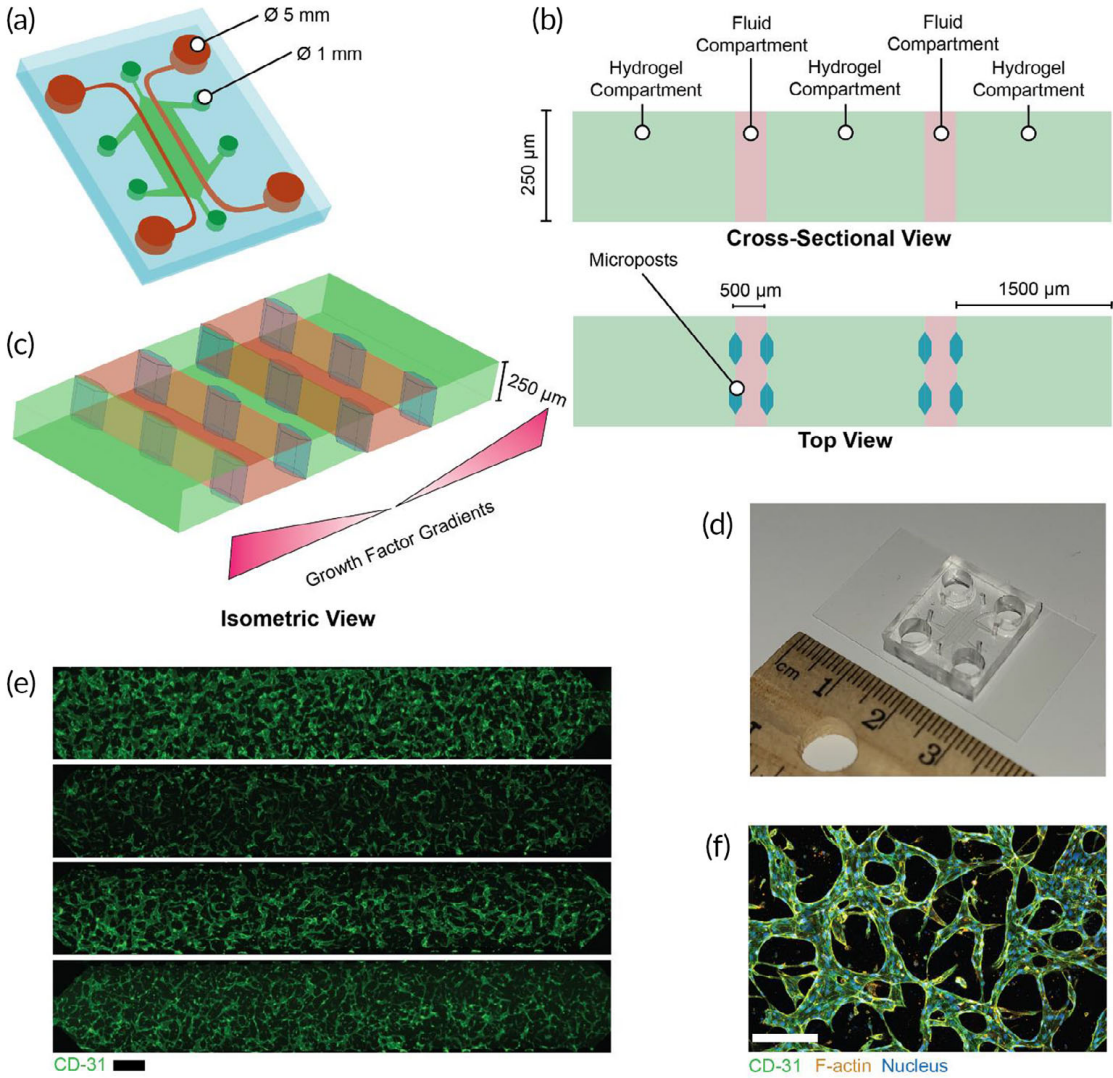
Design of vascularized microphysiological systems (vMPS) with control over biochemical and mechanical cues. (a) A 3D schematic of a vMPS consisting five channels supporting vasculogenesis. (b) A 2D cross‐section of the vMPS describing the resident tissue within the compartments of the vMPS, and (c) an isometric view showing microposts facilitating hydrogel‐fluid interfaces that generate growth factor gradients to a central channel, thus supporting vascular network formation. (d) A photograph of a vMPS used in this study, and (e) representative immunofluorescence images of vMPS consisting vascular networks formed via various combinations of factors that influence vasculogenesis. Scale bar = 500 μm. (f) Optically sectioned image of a vMPS. Scale bar = 250 μm.

We began this study by first generating 500 diverse samples of vMPS fabricated to a design that is relatively common in literature^20^, ^21^, ^22^, ^23^, ^24^, ^25^, ^26^ (Figure 1a–d) by selecting and varying EC source and density, fibroblast density, growth factor supplementation, and ECM stiffness—argued as the most critical determinants of vascular networks.^8^ Vascular networks formed within the vMPS were imaged and sections of approximately 8 × 1 mm (Figure 1e,f; Video S1) were morphologically analyzed (vessel coverage, vessel length, segment count, branchpoint count, mean segment length, mean segment diameter) or used to design machine learning algorithms. The oxygen flux and transport metrics through each of these networks were also measured computationally.^13^ From these data, we derived a normalized vascular oxygen concentration (OXY_*V*_) describing the area average intravascular oxygen concentration taking into consideration oxygen consumption by ECs, and a normalized tissue oxygen concentration (OXY_*T*_) describing the area average oxygen concentration delivered to the non‐vascular tissue of the vMPS. We quantified these two attributes because they together represent the oxygen transport function of vascular networks—oxygen shuttling through vascular networks and subsequent delivery to tissues. We applied these metrics (OXY_*T*_, OXY_*V*_, or both) as a measure of oxygen against which we evaluated the standard morphological measures of vascular networks as well as our machine learning models.

To evaluate our deep learning models, we employed routinely used statistical descriptors, for example: (1). the standard deviation of the residuals (root mean square error [RMSE]); (2) the average absolute value of the residuals (mean absolute error [MAE]), and (3) the proportion of the variance in the data that is explained by the model (*R* ^2^). RMSE and MAE values closer to zero and *R* ^2^ values closer to one indicate higher correlation and accuracy between measured and predicted values.

### 3.2 Chained neural network predicts a biological function of vascularized networks

**FIGURE 2:**
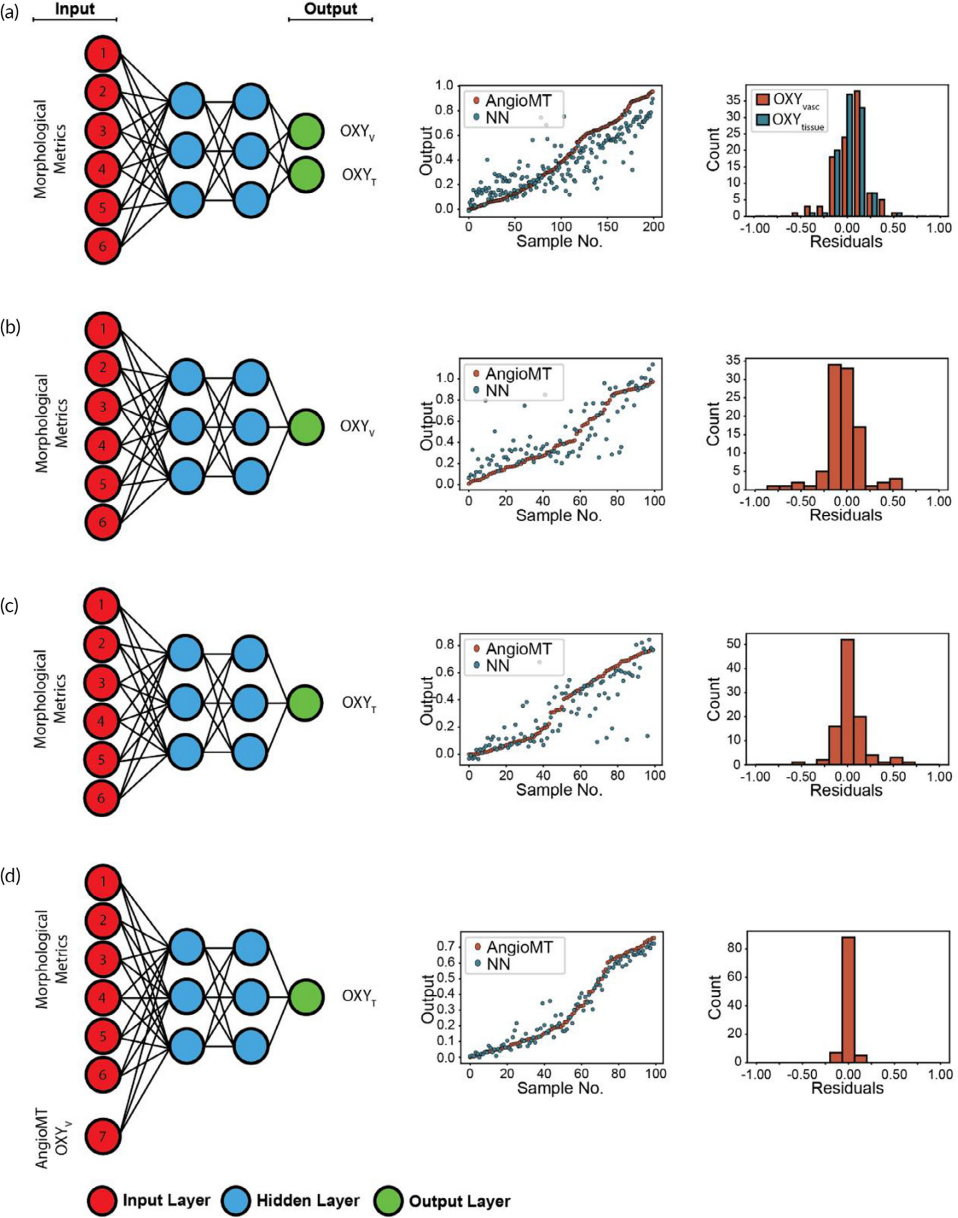
Neural network analyses of the vMPS. Infographic describing the input and output parameters (input, hidden, and output layers) of a neural network algorithm applied (left), graph describing relative error between measured values of AngioMT versus predictions from a neural network (NN, center), and residual plots (right) when the neural network is designed such that it predicts (a) a multi‐output—OXY_V_ and OXY_T_—from the six morphological metrics, (b) a single‐output—OXY_V_—from the morphological metrics, (c) a single‐output—OXY_T_—from the morphological metrics, and (d) a single‐output—OXY_T_—from the morphological data and OXY_V_ from AngioMT. vMPS, vascularized microphysiological systems.

With ground truth data split into an 80%–20% training–testing split and a testing scheme in hand, we next tested a multi‐output neural network taking only morphological data as input and predicting both OXY_*T*_ and OXY_*V*_ (Figure 2a). When compared to measured data from AngioMT, we found a normal distribution of residuals but modest accuracy and correlation (Figure 2a, Table 1, RMSE: 0.17, MAE: 0.13, *R* ^2^:0.67). Then, we tested single‐output models that predicted OXY_*V*_ (Figure 2b) and OXY_*T*_ (Figure 2c) independently and found a continued normal distribution of residuals around 0 but little change in accuracy or correlation (Figure 2b,c, Table 1, RMSE: 0.16 and 0.12, MAE: 012 and 0.17, *R* ^2^: 0.75 and 0.80, for neural networks predicting OXY_*V*_ and OXY_*T*_, respectively). Therefore, morphological metrics alone do not serve as an effective input of these neural networks.

**TABLE 1:** Neural network model architectures under investigation and their accuracy metrics following testing.

Model architectures	Multi‐output	Single‐output			Chained
Input	Morphology	Morphology	Morphology	Morphology + AngioMT OXY_V_ values	Morphology
Prediction accuracy metric	OXY_*V*_ and OXY_*T*_	OXY_*V*_	OXY_*T*_	OXY_*T*_	OXY_*T*_
*R* ^2^	0.67	0.75	0.80	0.98	0.88
MAE	0.13	0.12	0.07	0.02	0.06
MSE	0.03	0.02	0.01	0.00	0.01
RMSE	0.17	0.16	0.12	0.03	0.09

Abbreviations: MAE, mean absolute error; MSE, mean square error; RMSE, root mean square error.

We next hypothesized that the accuracy of a neural network predicting OXY_*T*_ from morphological data alone (Figure 2c) could be enhanced if there were an additional input representing the oxygen concentration diffusing into the tissue, described in AngioMT's OXY_*V*_ measurement. Therefore, we next performed an experiment using the OXY_*V*_ measure as an additional input with the original six morphological metrics as original inputs to a single‐output neural network predicting OXY_*T*_ (Figure 2d). Here, we found a significant increase in accuracy when quantifying the model's performance against the ground truth data (Figure 2d, Table 1, RMSE: 0.03, MAE: 0.02, *R* ^2^: 0.98). While this strategy appears to produce a highly effective neural network model, the calculation of the OXY_*V*_ parameter is highly resource‐intensive in practice and requires an explicit use of AngioMT computations, thus still resulting in a user‐dependent intervention. Therefore, there would be little practical advantage in applying this algorithm.

**FIGURE 3:**
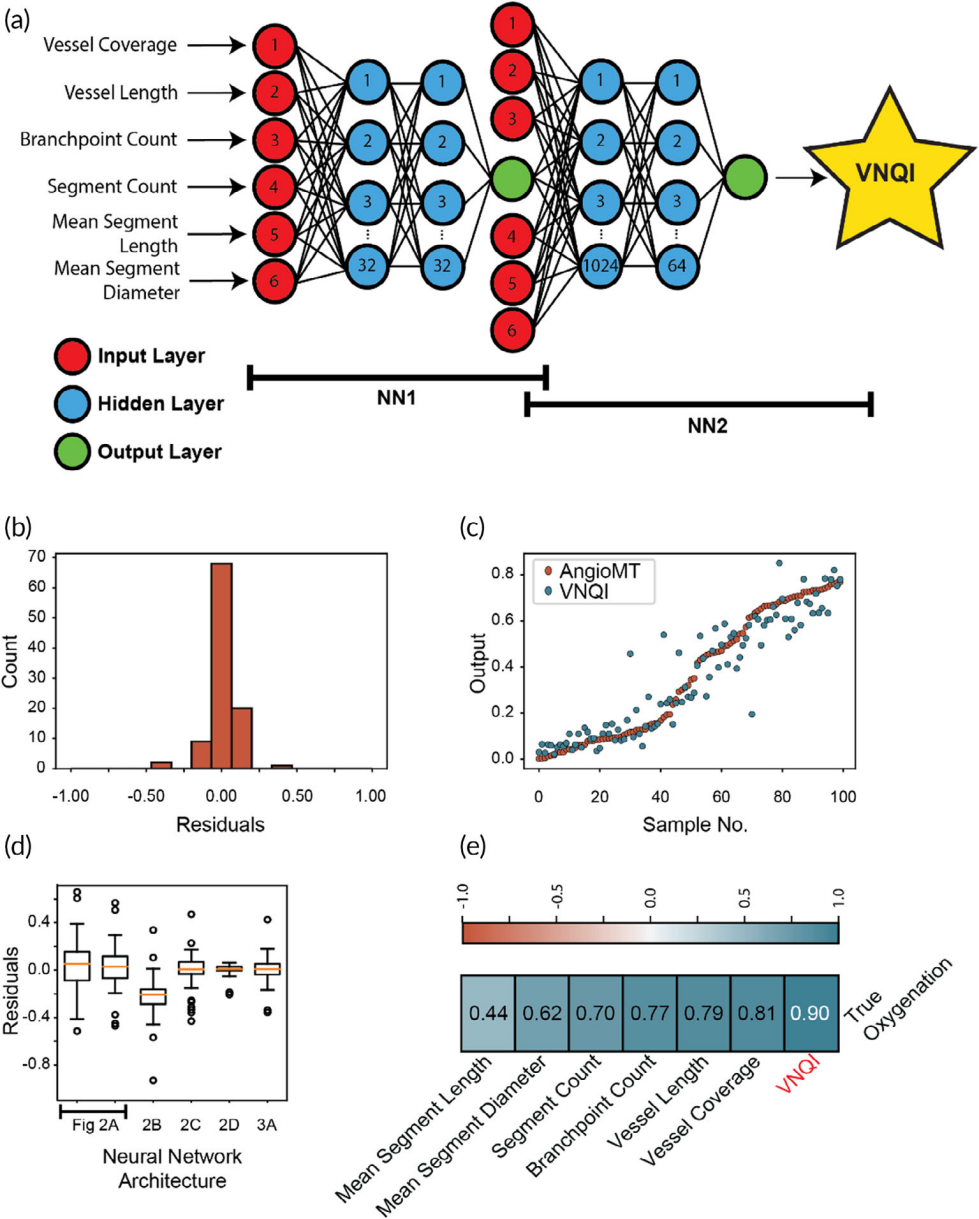
Chained neural network analysis of vMPS. (a) A schematic of the chained neural network algorithm architecture, resulting in a singular output as VNQI. (b) Histogram of the residuals calculated from the analysis of the VNQI from the training dataset. (c) Scatter plot showing AngioMT (measured) and VNQI (predicted) values for the test dataset. (d) Box plots comparing the residuals for all five neural network models (labeled with previous figure panels, yellow: mean; box: interquartile region; whiskers: minimum/maximum; circles: outliers) in this study (*n* = 100). (e) Pearson R Correlation Test of all inputs and VNQI against AngioMT measurements. vMPS, vascularized microphysiological systems; VNQI, vascular network quality index.

To address this challenge, we conceived an algorithm consisting of a chain of neural networks, where one network took morphological data alone as input and predicted OXY_*V*_ (NN1), and a second neural network (NN2) adopted the OXY_*V*_ prediction and morphological data to then predict OXY_*T*_ (Figure 3a). To establish this chain, the first model was trained using morphological data alone as input, while the second neural network was trained with morphological and AngioMT OXY_V_ data as input, and the two networks were joined in a chain. We evaluated the chained model with the testing data similar to the previous models, and found significantly improved accuracies and correlations relative to the neural networks using morphological data as input alone (Figure 3b,c, Table 1, RMSE: 0.09, MAE: 0.06, *R* ^2^:0.88). Further, analysis of each of the neural networks tested in this study showed that the chained neural network had similar ranges and outlier proportions to the neural network trained with measured OXY_*V*_ data, whereas others trained with morphological data as input alone had higher residual ranges and proportions of outliers (Figure 3d). This additionally suggested that accurate predictions of OXY_*T*_ rely on knowledge of the available oxygen that can diffuse into the tissue (OXY_V_), and OXY_T_ cannot be accurately predicted using morphology alone. Therefore, tissue oxygenation may largely be dependent on the available oxygen from the vasculature.

Since our chained neural network (NN1 and NN2) outperformed all other iterations of the model, the output of this model was evaluated and named as the VNQI. A Pearson R correlation test between all standard morphological metrics as well as VNQI against the measured oxygen revealed that VNQI correlated to measured data to the highest extent relative to all other commonly used metrics (Figure 3e). Therefore, VNQI may serve as a machine‐learned metric that can be applied to a physiologically‐relevant and user‐independent objective evaluation of vascularized networks without requiring extensive experimental or computational resources.

Finally, we performed post hoc analyses to determine the adequacy of this deep learning work relative to prior used regression models, as well as to verify that the sample size used in this study was sufficient to generate a reliable and stable model. The accuracy metrics of the chained neural metric exceeded the statistical scores against regression models (Table S1). To gain more insight into the stability and accuracy of our network model, we calculated the mean and standard deviation of each of the neural networks making up the chained architecture. Here, we saw that with 10 different partitionings of the dataset, standard deviations for each statistical metric remained very close to 0 (Table S2). Lastly, to examine if our training set size was appropriate, we examined how our model performed as we increased the training set size. We found that after 300 samples, the error for the training and validation sets became steady, suggesting that our dataset of 500 images is sufficient (Figure S2).

### 3.3 VNQI is sensitive to determinants of vascular network formation in engineered biosystems

In practice, engineered biosystems are manufactured with a variety of different ECs with variable cell densities, supporting proangiogenic perivascular cells (e.g., fibroblasts), and growth factor cocktails. These combinations often depend upon prior protocols and experience among different laboratories, and in some cases, depending upon the specific biological question being asked.^8^ Since no standard practice for vascularizing and subsequently evaluating the vascular network quality exist, we challenged the VNQI to assess if it may contribute to the standardization and characterization of the several techniques used to build such systems with desirable physiological relevance.

#### 3.3.1 Extracellular matrix

**FIGURE 4:**
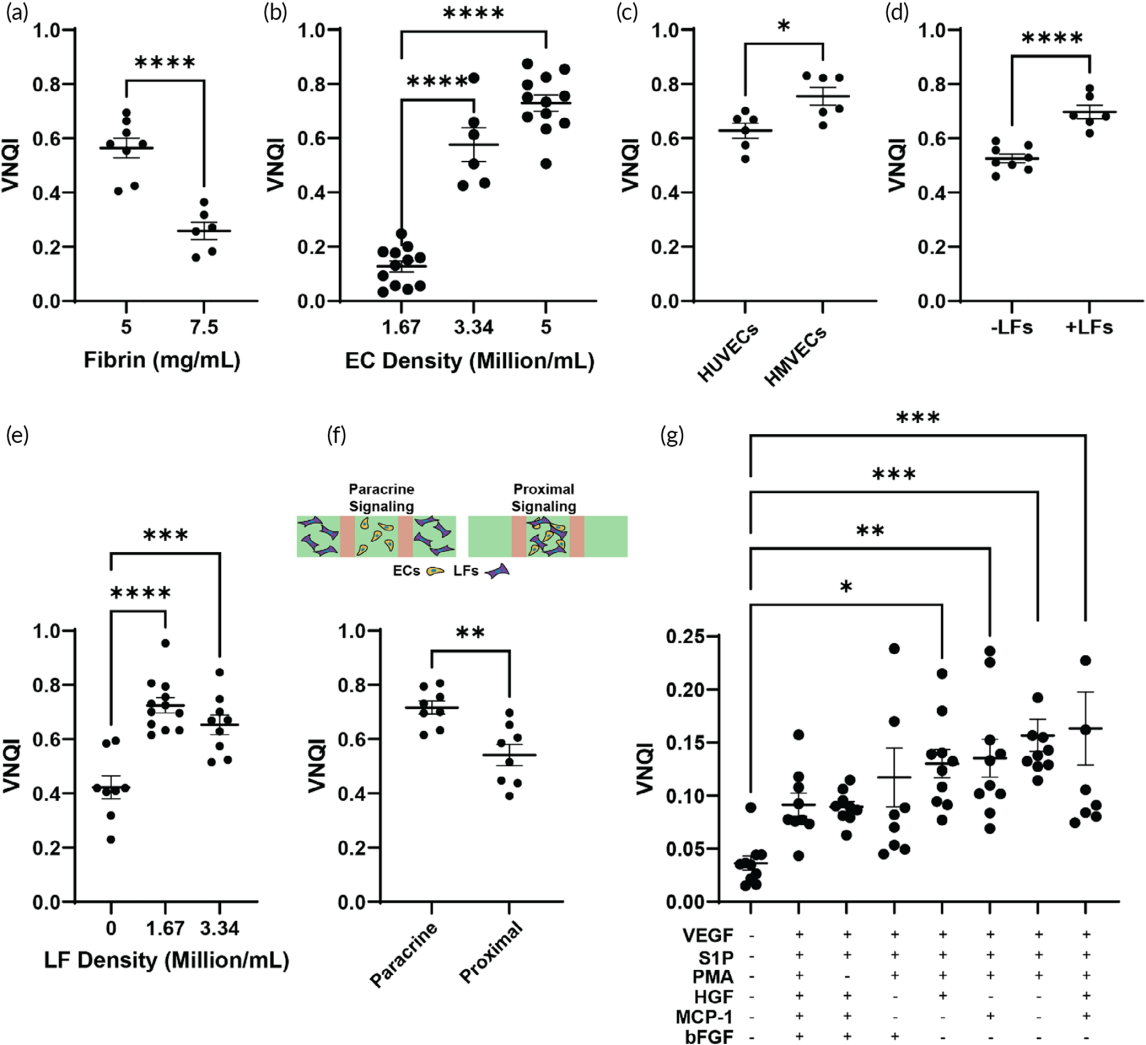
Sensitivity analyses of machine‐learning based VNQI score in the assessment of vascular networks. Change in VNQI upon varying the (a) fibrin matrix concentration (stiffness) (sample size: 5 mg/mL: *n* = 8, 7.5 mg/mL: *n* = 6; two‐tailed unpaired *t*‐test), (b) endothelial cell (HUVEC) density (sample size: 1.67: *n* = 12, 3.34: *n* = 6, 5: *n* = 12; one tailed ordinary ANOVA test), (c) endothelial cell‐type (HUVECs vs. HMVEC) (*n* = 6; two‐tailed unpaired *t*‐test), (d) co‐culturing ECs with perivascular lung fibroblasts (LFs) (sample size: +LF: *n* = 6, ‐LF: *n* = 8; two‐tailed unpaired *t*‐test), (e) LF density (sample size: 0: *n* = 8, 1.67: *n* = 12, 3.34: *n* = 10; one tailed ordinary ANOVA test), (f) LF compartmentalization inducing paracrine or proximal EC‐LF signaling (*n* = 8; two‐tailed unpaired *t*‐test), and (g) growth factor cocktail (*n* = 10; one tailed ordinary ANOVA test). VNQI, vascular network quality index. Significance is reported as *p < 0.05; **p < 0.01; ***p < 0.001; ****p < 0.0001.

Matrix remodeling due to biological processes and signaling (such as in the tumor microenvironment^27^, ^28^) may influence the vascular network architecture, and thus the vasculature's potential to oxygenate. However, it is unclear if previously established vascular quality metrics also correlate to tissue oxygenation due to ECM modification. When we tested varying hydrogel (fibrin) stiffnesses in our vMPS (concentration: 5 and 7.5 mg/mL corresponding to compressive moduli:1.5 or 9 kPa and porosities: 9% or 26%), we found that the resultant vascular network architecture was distinct, where higher concentrations (i.e., stiffer and less porous matrices) resulted in relatively sparse vascularization with less oxygenation, as described by the VNQI (Figure 4a).

#### 3.3.2 Endothelial cell density and type

Next, we examined the vascular networks formed by varying the two most commonly used ECs: HUVECs and HMVECs.^8^ First, we examined vMPS containing increasing concentrations of ECs and observed that VNQI increased with EC density (Figure 4b), as has been observed in prior experimental systems.^24^ Next, we tested the two variable EC types and found that HMVECs scored higher than HUVECs (Figure 4c). This outcome is consistent with prior comparisons showing HMVECs have a relatively higher angiogenic potential relative to HUVECs, because of their potential to release more VEGF than HUVECs.^29^

#### 3.3.3 Perivascular cells

More recent vMPS are designed to include cell cocultures, where ECs may be cocultured with perivascular cells, for example, lung fibroblasts (LFs) that often influence vascularization dynamics (e.g., in wound healing, LFs and ECs are often recruited to fibrin clots to revascularize the tissue^30^, ^31^). When we engineered a separately compartmentalized EC‐LF coculture model (i.e., the two cell types do not directly contact each other), we found that VNQI correspondingly differed based on the presence or absence of these perivascular cells (Figure 4d). This reveals that the presence of LFs may enhance biological performance of vascular networks.

Since prior reports are also inconsistent with fibroblast cell density, and it may be expected that in practice, seeding density of these cells will influence the outcomes of the vascularized networks, we also set out to perform a sensitivity test of VNQI against fibroblast seeding density. We found that when we seeded vMPS with 5 million HUVECs/mL cocultured with fibroblasts of increasing concentration in separate compartments, the density of the fibroblasts influenced the vascular network's VNQI score (Figure 4e). In particular, while the presence of LFs in all tested densities resulted in an improved VNQI, we saw a minor and statistically insignificant decrease in VNQI between the 1.67 and 3.34 million LF/mL groups. This small change or saturation may be possible as higher LF density could result in secretion of higher or competing amounts of matrix metalloproteinases (MMPs) that break down the fibrin hydrogel faster.

Besides ECs and fibroblasts being cultured in separate compartments (paracrine signaling), some reports also describe culturing the fibroblasts or other stromal cell types in the same hydrogel channel as the ECs (proximal signaling, Figure 4f). We compared the impact of proximal signaling LFs against paracrine signaling LFs in an experiment where the total number of fibroblasts were kept consistent (3.34 million LFs/mL in a single central channel of proximal signaling vMPS, 1.67 million LFs/mL in two outer channels of paracrine signaling vMPS). We found that proximally signaling LFs had a significantly lower VNQI relative to paracrine signaling, suggesting that vMPS designs can be optimized or fabricated with various LF arrangements using VNQI as an endpoint measurement.

#### 3.3.4 Growth factors

Finally, since almost every biosystem is engineered using growth factor cocktails, we set out to test the sensitivity of VNQI to vMPS grown with differential growth factor cocktails. Here, we determined a testing scheme based on a previous review of angiogenesis literature^8^ resulting in seven different growth factor cocktails consisting of six different factors (see Section 2). We first saw that growth factor cocktails containing all factors, or all factors minus PMA, only scored slightly increased VNQI to conditions where no growth factors were added (Figure 4g). The group with all growth factors could have led to overstimulation, similar to the experiment finding higher LF concentrations slightly decreased VNQI measurements. Next, we saw that four groups scored significantly higher than the control. The commonality between these was the inclusion of VEGF, S1P, and PMA, plus one or both of HGF and MCP‐1, and omission of bFGF. While a similar group having only VEGF, S1P, PMA, and bFGF had a trivially lower VNQI than these groups, the consistently high score of vMPS without bFGF suggests that this pro‐angiogenic factor might reduce vasculogenesis despite being a potent pro‐angiogenic factor, and could thus warrant further investigation in subsequent studies.

**FIGURE 5:**
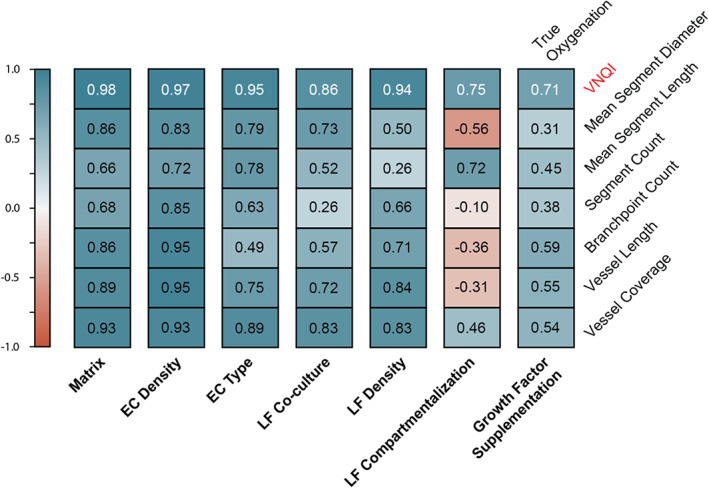
Pearson R correlation of VNQI and morphological metrics against measured oxygen. Cross‐correlation of VNQI and standard metrics against AngioMT measures shown for each individual experimental group that tested the sensitivity of the VNQI upon varying the vMPS by imposing differential biochemical or mechanical conditions. VNQI, vascular network quality index.

With experiments testing different biophysical and biochemical elements of vMPS in hand, we next set out to further demonstrate the validity of VNQI by performing a Pearson R correlation test for each experimental group. We found that in each case, VNQI correlated with the measured data to a higher extent than any of the six morphological metrics currently used (Figure 5). Taken together, VNQI serves as a more physiologically‐relevant metric that may be used to design, characterize, and evaluate vascularized networks and their biological responses to chemical and mechanical stimuli.

### 3.4 vMPS engineered and optimized by VNQI predict state of disease and evaluate treatment

Successful vascularization during cell transplantation therapy is essential to sustain and improve transplant health and survival, which positively influences patient outcomes.^32^, ^33^, ^34^ For example, when using the Edmonton Protocol, islets co‐transplanted with vascular ECs and pericytes or hMSCs have been shown to improve the disease outcome of T1D patients.^33^ While it is known that the available tissue oxygen content directly determines the success rate of a transplantation treatment, there are no effective quantitative measures to evaluate the success of these transplants both in preclinical or clinical phases. Therefore, we set out to ultimately evaluate VNQI's capability by measuring the state of health of a vascularized islet transplantation model.

**FIGURE 6:**
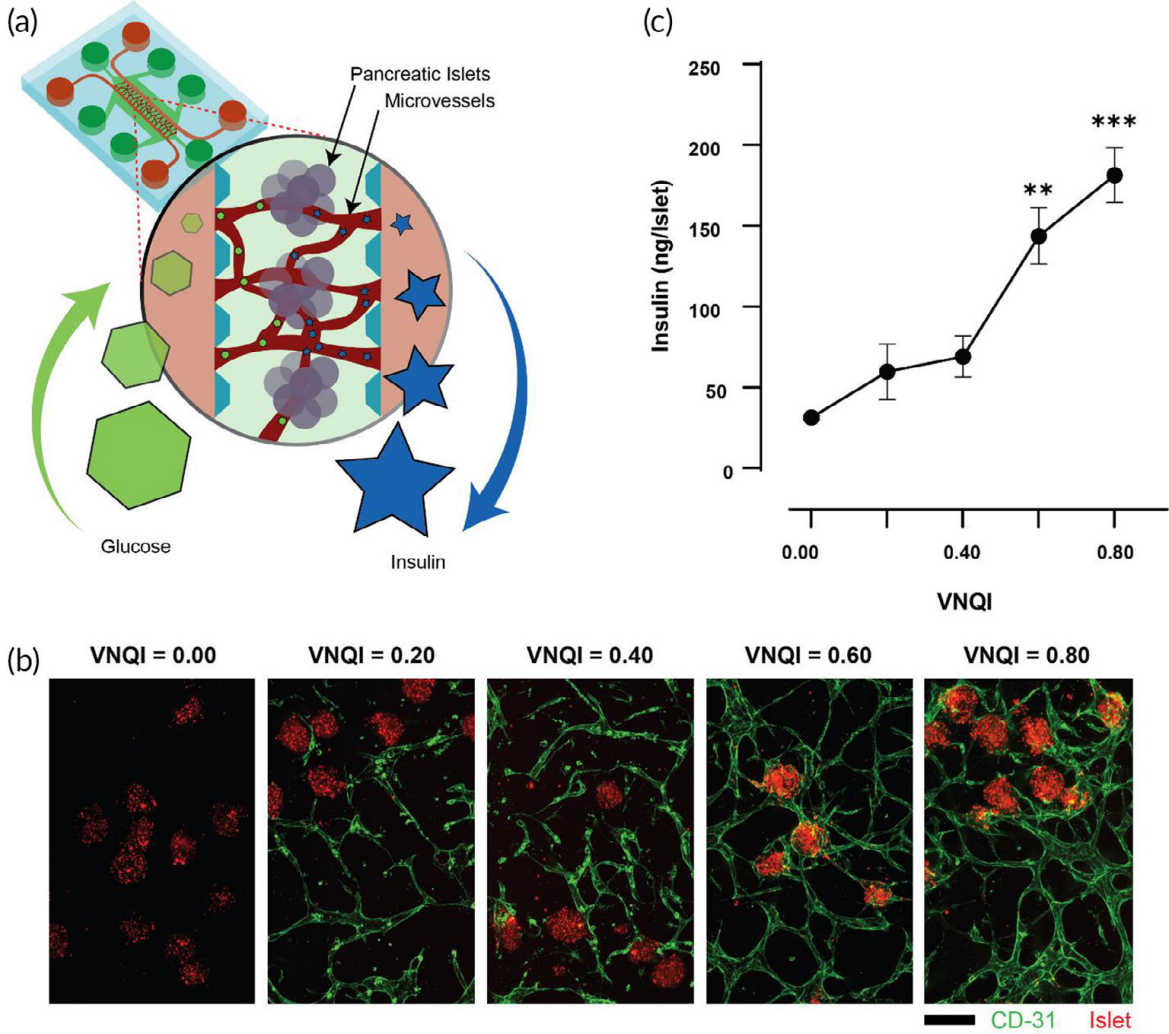
Analysis of a cell transplant preclinical model with vascularized islet‐chips designed using the VNQI. (a) Schematic a functional (glucose‐responsive) vascularized islet‐chip. (b) Immunofluorescence images of vascularized islet‐chips manufactured with varying the VNQI regulated by mechanical and chemical determinants of vasculogenesis (scale bar = 125 μm). (c) Islet insulin secretion after hypoxia challenge upon increasing VNQI (*n* = 3; one tailed ordinary ANOVA test with comparisons to VNQI = 0 control only). VNQI, vascular network quality index. Significance is reported as *p < 0.05; **p < 0.01.

We first designed and engineered our own vascularized islet‐chip model (Figure 6a) because protocols describing islet‐chips that support vascular networks are sparse. We used VNQI as a design criterion to control the vasculature surrounding islet spheroids and manufactured vascularized islet‐chips with variable oxygen‐carrying capacities, scored by VNQI (Figure 6b; Video S2). Having a fully vascularized islet‐chip, we next hypothesized that the degree of vascularization will determine the functionality of the chip if it were to undergo physiologically relevant challenges, such as hypoxia. Since islet function depends on its capability to secrete insulin, which is directly impacted by the availability of oxygen delivered by vasculature to the islets, we used insulin as the functional readout of our measure of islet health. Further, many reports show that hypoxia decreases insulin secretion in islets or β‐cells both in vitro and in vivo.^35^, ^36^, ^37^ Therefore, we engineered vascularized islet‐chips of increasing VNQI (Figure 6c), and after the standard culturing period, we subjected each chip to a hypoxia challenge while being perfused with oxygen‐ and glucose‐rich media. Indeed, we found that islets containing a vascular network of increasingly high VNQI maintained and improved insulin secretion compared to islets with poor or non‐existent vasculature (Figure 6d). This indicates that VNQI may assist in designing preclinical models of vascularized islets and may serve as a quantitative tool to evaluate cell replacement therapies that are dependent on the extent of vascularization.

## 4 DISCUSSION

Recent advances describing vascularization of in vitro preclinical models have enabled nutrient and gas exchange as well as waste elimination from tissues, thus improving these models' biological mimicry of organ‐level functions.^4^, ^8^ In parallel, artificial intelligence and/or machine learning (AI/ML) applications to MPS have been an exciting development with tremendous potential to characterize cell–cell interactions or diagnose a state of health, as already presented in a few recent studies.^38^ By employing an AI/ML approach to quantitatively evaluate preclinical models of vascularized biosystems, this study describes a broadly applicable and user‐independent quantitative metric, VNQI, to analyze capillary networks as they develop and perform their basic function of supplying oxygen to the resident tissue. Importantly, VNQI's sensitivity to the critical mechanical and chemical determinants of vasculogenesis (Figures 4 and 5) will help shift the *status quo* of using relatively simple and unvalidated morphological metrics toward a singular, automated, and validated metric.

Since VNQI correlates to the oxygen capacity of a vascular network in both vessels and tissues, our parametric investigations of the determinants of vasculogenesis (for example, matrix stiffness, EC type, perivascular cells, etc.) may also inform the relationship of these factors to tissue oxygenation and nutrition. As an example, when we replace LFs with exogenously supplied growth factors, bFGF limited vasculogenic network formation, despite some previous reports highlighting the potency of bFGF in angiogenesis assays.^39^, ^40^ Finally, we could observe and characterize matrix stiffness and its influence on tissue oxygenation, that could help in our ability to study mechanotransduction and its translational capacity in complex tissues and diseases.

Our use of an area average oxygen concentration for the 1 × 1 mm images in this work was to focus on a practical way to assess a vascular network in a tissue model with a singular metric. As a result, it allowed us to build the normalized and dimensionless metric VNQI which scales from 0 (low average oxygen) to 1 (high average oxygen). However, a vascularized tissue has a heterogenous oxygen gradient, and the vascular function may be regulated by the local microenvironment and oxygen tension. This detail is not captured fully by the neural network, but it can be derived from AngioMT software.

Ultimately, our study shows that integrating AI/ML approaches can help design even more complex multicellular systems, such as a pancreatic microenvironment or islet‐chips undergoing dynamic changes such as oxygen depletion or hypoxia. For example, by designing islet‐chips based on the VNQI score, we selected combinations of mechanical and chemical conditions that resulted in islet‐chips of variable insulin secretion, which decreased the number of chips, reagents, islets, and ELISA assays in our in vitro screening. From our screening of these chips, we could present a proof‐of‐concept of optimizing vascularized transplant systems preclinically. In summary, since the calculation of the VNQI is rapid and requires moderate computational resources, this quantification strategy is expected to propel organ‐chips into situations where large samples of vascularized constructs, grafts, or cell therapies are screened in vitro before costlier in vivo studies.

## AUTHOR CONTRIBUTIONS

**James J. Tronolone:** Formal analysis (lead); investigation (lead); methodology (lead); writing – original draft (equal). **Tanmay Mathur:** Methodology (supporting); software (lead). **Christopher P. Chaftari:** Investigation (supporting). **Yuxiang Sun:** Resources (supporting). **Abhishek Jain:** Conceptualization (lead); funding acquisition (lead); supervision (lead); writing – original draft (equal).

## CONFLICT OF INTEREST STATEMENT

The authors declare no conflicts of interest.

### PEER REVIEW

The peer review history for this article is available at https://www.webofscience.com/api/gateway/wos/peer-review/10.1002/btm2.10582.

## Supporting information


**VIDEO S1.** Rotating Z‐stack confocal image of a vMPS. Green—CD‐31; orange—F‐actin; blue—nucleus.

**VIDEO S2.** Rotating Z‐stack confocal of a vascularized islet‐chip. Red—islets; green—CD‐31.

**DATA S1:** Supporting information.

## Data Availability

Images of the vMPS, as well as python code for training the neural networks and for calculating VNQI are available upon request to the corresponding author.
