# Supplementary material for: Machine learning chained neural network analysis of oxygen transport amplifies the physiological relevance of vascularized microphysiological systems
Source: Bioeng Transl Med. 2023 Aug 1;8(6):e10582. doi: 10.1002/btm2.10582 (PMC10658488; doi:10.1002/btm2.10582)
Supplement: Supplementary file 3 — DATA S1: Supporting information. [file BTM2-8-e10582-s002.docx]

**Machine learning chained neural network analysis of oxygen transport amplifies the physiological relevance of vascularized microfluidic systems**

James J. Tronolone^1^, Tanmay Mathur^1^, Christopher P. Chaftari^1^, Yuxiang Sun^2^, Abhishek Jain^1, 3, 4*^

**SUPPLEMENTARY INFORMATION**

**Table S1** – Accuracy metrics for various multi-variate regression methods

| *_Regression Model_  ^Accuracy Metric^* | *R^2^* | *MAE* | *MSE* | *RMSE* |
| --- | --- | --- | --- | --- |
| **Multiple linear regression (MLR)** | 0.74 | 0.09 | 0.01 | 0.13 |
| **MLR with recursive feature elimination with cross-validation** | 0.74 | 0.09 | 0.01 | 0.13 |
| **MLR with engineered features via PCA** | 0.71 | 0.10 | 0.02 | 0.14 |
| **Decision tree** | 0.55 | 0.12 | 0.04 | 0.20 |
| **Pruned decision tree** | 0.61 | 0.13 | 0.30 | 0.20 |
| **Random forest** | 0.70 | 0.11 | 0.02 | 0.16 |
| **Chained neural network** | 0.88 | 0.06 | 0.01 | 0.09 |

**Table 2** – Accuracy metrics for each part of the chained algorithm, reported as mean ± standard deviation

| *_Accuracy Metric_  ^Neural Network^* | *First Network* | *Second Network* | *Complete Chain* |
| --- | --- | --- | --- |
| **R^2^** | 0.75 ± 0.05 | 0.98 ± 0.01 | 0.88 ± 0.06 |
| **MAE** | 0.12 ± 0.01 | 0.02 ± 0.01 | 0.06 ± 0.01 |
| **MSE** | 0.02 ± 0.01 | 0.001 ± 0.001 | 0.01 ± 0.01 |
| **RMSE** | 0.16 ± 0.02 | 0.03 ± 0.01 | 0.09 ± 0.02 |

**
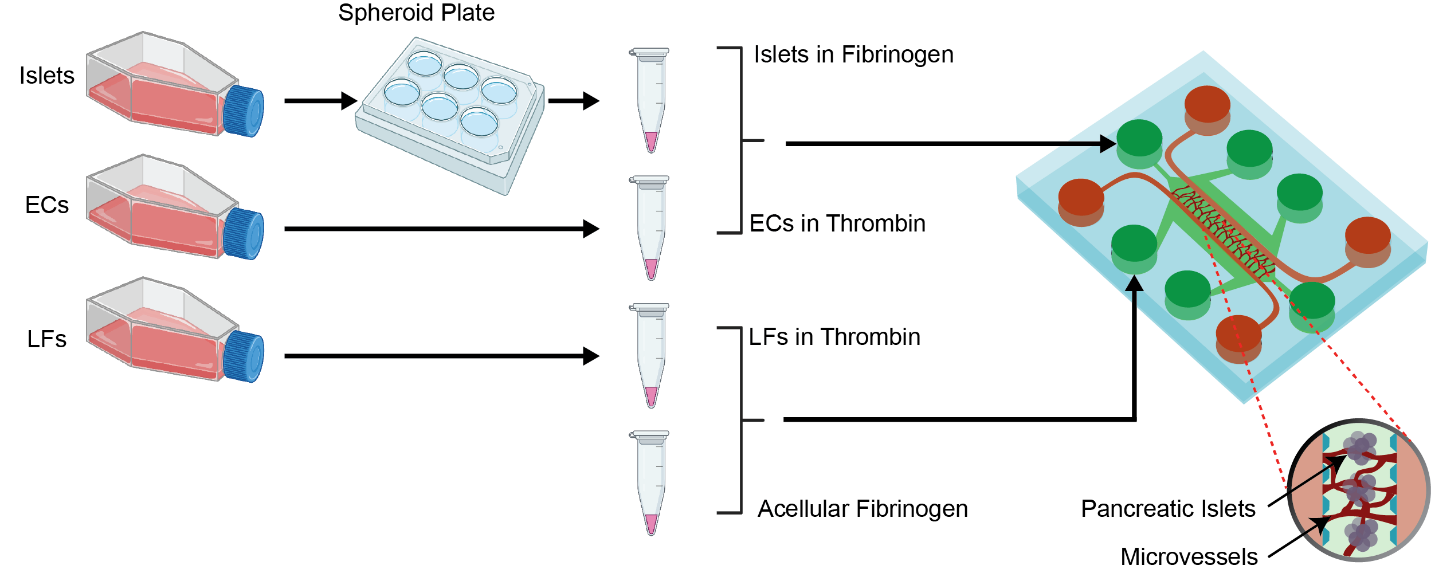
**

**Figure S1 –** Schematic showing the protocol for engineering the vascularized islet-chip.


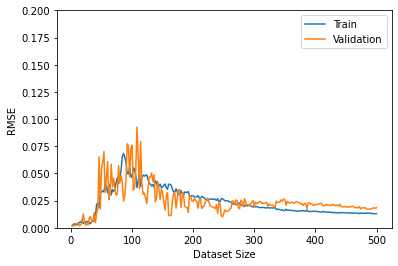


**Figure S2** – Train and validation performance (RMSE) as a function of dataset size for the chained neural network algorithm

**VIDEOS**

**Video S1.** Rotating Z-stack confocal image of a vMPS. Green – CD-31; orange – F-actin; blue – nucleus.

**Video S2.** Rotating Z-stack confocal of a vascularized islet-chip. Red – islets; green – CD-31.
